# Adolescent suicide attempts in three diverse island nations: patterns, contextual differences and demographic associations

**DOI:** 10.1186/s13104-021-05804-4

**Published:** 2021-12-23

**Authors:** Jinrong Fu, Olumide Abiodun, Michael Lowery Wilson, Masood Ali Shaikh

**Affiliations:** 1 Centre for Injury Prevention and Community Safety (CIPCS), PeerCorps Trust Fund, 352/64 Makunganya Street, Co-Architecture Building, 4th Floor, P.O. Box 22499, Dar es Salaam, Tanzania; 2 ICT University, 1 Avenue Dispensaire Messassi, Zoatoupsi, Yaounde, Cameroon; 3 Heidelberg Institute of Global Health, Heidelberg University, Heidelberg, Germany

**Keywords:** Adolescent health, Epidemiology, Self-harm, Global health, Mental health, School health

## Abstract

**Objective:**

Most epidemiological studies on suicidal behavior have been focused on high-income country settings. This study examine factors associated with suicidal behaviors among school-attending adolescents in three island nations. In this secondary analysis of the publicly available 2015 nationally representative GSHS data, we tested demographic, social, and behavioral attributes using multivariable logistic regression to association with suicide attempts.

**Results:**

Within the recall period, 13.6% of participants reported having attempted suicide one or more times in the Cook Islands, 10.8% in Curaçao, and 9.8% in East Timor. In the Cook Islands, suicide ideation (AOR = 19.42, 95% CI = 9.11–41.41), anxiety (AOR = 2.51, 95% CI = 1.08–5.82), physical bullying (AOR = 3.3, 95% CI = 1.10–9.91), and cigarette smoking (AOR = 3.82, 95% CI = 1.38–10.54) were associated with suicide attempts. For Curaçaoo, suicide ideation (AOR = 7.88, 95% CI = 5.20–11.95) and suicide planning (AOR = 7.01, 95% CI = 4.24–11.60) were statistically significant. While for East Timor, suicide ideation (AOR = 4.59, 95% CI = 2.14–9.88), suicide planning (AOR = 3.36, 95% CI = 1.76–6.29), bullying victimization (AOR = 2.69, 95% CI = 1.02–7.12), and serious injuries (AOR = 2.22, 95% CI = 1.31–3.74) were statistically significant. Suicide attempt is relatively common in each of the three island nations. The socioeconomic context of adolescents might play a significant role in moderating suicidal behavior. Therefore, prevention efforts should be grounded in view of geographic, demographic, and socioeconomic contexts of the populations at risk.

**Supplementary Information:**

The online version of this article 10.1186/s13104-021-05804-4 contains supplementary material, which is available to authorized users.

## Introduction

Suicide claims the lives of more than 800,000 people globally each year [1, 2]. Among adolescents aged 15–19 years old, suicide is the third leading cause of death. While data from low- and middle-income countries (LMICs) typically underestimate the problem, available evidence suggests that the consequences of adolescent suicide attempts (SA) are prevalent in LMICs, where over 79% of global suicide deaths in 2016 occurred [2]. While numerous studies regarding suicidal behavior (SB) among adolescents have been done in mostly high-income western and Asian countries with large domestic populations [3–8], few studies exist from LMIC island nations.

The populations of the islands in our study—Cook Islands, Curaçao, and Timor-Leste are 17,564, 164,093 and 1,318,445, respectively, as of 2020 [9]. Persons aged under 24 years make up significant proportions of the overall populations—34.82% (Cook Islands), 33.06% (Curaçao), with Timor-Leste having a very young population overall with 60.28% [10–12]. Island nations that are not particularly subject to high rates of migration inflows may represent useful contexts in which to examine socio-behavioral phenomena.

In Curaçao, the number of victimizations reported by in school adolescents was associated with both mental health and health risk behaviors [13]. In Timor-Leste, suicidal behavior among school attending adolescents has been previously associated with unintentional injuries [14]. In the Cook Islands there have been calls for the prioritization of mental health and suicide prevention research both of which remain underfunded [15]. The present study aimed to examine the social and demographic correlates of suicidal behaviors among school-attending adolescents in three island settings.

## Main text

Publicly available data from nationally representative surveys conducted in the year 2015, from three island countries of Cook Islands, Curaçao, and East Timor were used for secondary analyses. Detailed information on the data collection methods, questionnaire procedures, response rates and data is available at http://www.cdc.gov/gshs/. Information was missing for age, sex, and both age and sex, for 3, 5, and 2 records, respectively in the Cook Islands; 10, 27, and 3 records in Curaçao; and 74, 202, and 57 records in East Timor. No cases were excluded to ensure a correct design-based analysis.

SA as a dependent variable was derived from a question in the GSHS: “during the past 12 months, how many times did you actually attempt suicide?” For this analysis, participants were classified as having attempted suicide if they reported having attempted one or more SA during the recall period. If no SA was reported, participants were classified as not having attempted suicide; for 3 records in the Cook Islands, 59 records in Curaçao, and 29 records in East Timor, this information was missing. Fifteen independent variables at the individual level were considered (age, sex, suicide ideation, suicide planning, anxiety, loneliness, bullying victimization, physical bullying victimization, involvement in physical fights, serious injury, early sexual debut, alcohol use, physical attack, marijuana use, and cigarette smoking), and six at the social level (presence of supportive parental figures, presence of helpful peers, the extent of the social network, parental smoking status, people smoked in presence, and food insecurity). Cook Islands GSHS 2015, did not ask questions about sexual experiences. Details on variable creation appear in supplement (Additional files 1, 2: Tables S1, S2).

Differences between SA involvement among the variables were screened for statistical significance using the survey version of the chi-square test, which is a design-adjusted version of Pearson’s chi-square test for categorical variables, and the design-adjusted version of the t-test for continuous variables (age and number of friends). We then created two binary logistic regression models. These were intended to model the ability of the selected independent variables to predict the dichotomized SA variable. The first model adjusted only for age and sex. While the second model included all variables significant at the bivariate level. We report the measures of association as adjusted (aOR) and unadjusted (OR) odds ratios along with 95% Confidence intervals (CI). Stata 16 (StataCorp, 2019) was used for analysis. All proportions—expressed in percentages—are weighted.

## Results

Within the recall period, 13.6% (unweighted count: 98) of participants reported having attempted suicide one or more times in the past 12 months in the Cook Islands, 10.8% (unweighted count: 296) in Curaçao, and 9.8% (unweighted count: 377) in East Timor. In the Cook Islands and Curaçao, most suicide attempters were female (53.4% and 61.0%, respectively). While in East Timor males comprised 55.7% of suicide attempters.

Table 1 shows the weighted distribution of selected factors according to suicidal behavior. The bivariate analyses show that in the Cook Islands, age, sex, food deprivation, parental tobacco use, people smoked in presence, and supportive parental figures were not statistically significantly associated with involvement in physical fights. In Curaçao, age and physical bullying were not statistically significant. In East Timor, age, sex, people smoked in presence, supportive parental figures, and helpful peers were not statistically significant.

**Table 1:** Comparison of factors by suicide attempt status in school-attending adolescents in Cook Islands, Curaçao, and Timor Leste, GSHS 2015

Variable	Cook Islands	Curaçao	East Timor
Age (SD)			
No suicide attempt	15.31 (1.42)	15.50 (1.90)	15.72 (1.80)
One or more suicide attempts	15.56 (1.46)	15.26 (1.70)	15.46 (2.00)
P-value	0.105	0.066	0.075
Sex (male)			
No suicide attempts	48.88	49.83	50.20
One or more suicide attempts	46.65	39.01	55.69
P-value	0.615	0.002	0.060
Suicide ideation			
No suicide attempts	7.93	6.06	6.55
One or more suicide attempts	64.62	54.91	42.14
P-value	< 0.001	< 0.001	< 0.001
Suicide planning			
No suicide attempts	8.43	4.09	6.20
One or more suicide attempts	55.73	50.23	45.20
P-value	< 0.001	< 0.001	< 0.001
Anxiety			
No suicide attempts	11.13	9.26	11.54
One or more suicide attempts	36.18	29.19	22.53
P-value	< 0.001	< 0.001	< 0.001
Loneliness			
No suicide attempts	6.06	12.79	13.85
One or more suicide attempts	22.95	37.83	24.36
P-value	< 0.001	< 0.001	< 0.001
Food deprivation			
No suicide attempts	9.29	2.96	11.10
One or more suicide attempts	14.94	10.88	18.13
P-value	0.096	< 0.001	< 0.001
Close friends (SD)			
No suicide attempts	2.67 (0.79)	2.21 (1.00)	2.54 (0.83)
One or more suicide attempts	2.28 (1.11)	1.96 (1.11)	2.30 (1.01)
P-value	0.024	0.001	0.001
Bullying victimization			
No suicide attempts	7.27	8.63	5.11
One or more suicide attempts	29.72	26.22	24.98
P-value	< 0.001	< 0.001	< 0.001
Physical bullying			
No suicide attempts	2.78	0.68	3.74
One or more suicide attempts	10.98	1.93	14.28
P-value	< 0.001	0.055	< 0.001
Parental tobacco use			
No suicide attempts	42.72	19.8	30.91
One or more suicide attempts	45.13	32.7	44.75
P-value	0.626	< 0.001	< 0.001
People smoked in presence during the last week			
No suicide attempts	43.84	26.04	45.39
One or more suicide attempts	50.48	36.66	47.04
P-value	0.23	< 0.001	0.503
Physical fight			
No suicide attempts	11.41	6.75	12.04
One or more suicide attempts	34.82	21.2	30.47
P-value	< 0.001	< 0.001	< 0.001
Serious injury			
No suicide attempts	50.88	29.26	66.73
One or more suicide attempts	71.64	49.29	89.51
P-value	< 0.001	< 0.001	< 0.001
Early sexual debut			
No suicide attempts	Not asked	21.72	7.37
One or more suicide attempts		35.58	18.55
P-value		< 0.001	< 0.001
Alcohol use in the past 30 days			
No suicide attempts	33.38	38.84	14.62
One or more suicide attempts	50.87	57.87	41.13
P-value	0.028	< 0.001	< 0.001
Physically attacked			
No suicide attempts	17.69	6.78	18.51
One or more suicide attempts	35.00	22.22	33.10
P-value	< 0.001	< 0.001	< 0.001
Marijuana use in the past 30 days			
No suicide attempts	3.73	4.70	3.45
One or more suicide attempts	17.92	12.56	23.58
P-value	< 0.001	< 0.001	< 0.001
Smoked cigarettes			
No suicide attempts	17.42	7.50	20.51
One or more suicide attempts	37.41	20.86	43.94
P-value	0.006	< 0.001	< 0.001
Supportive parental figures			
No suicide attempts	29.14	54.94	11.46
One or more suicide attempts	25.62	38.00	14.90
P-value	0.473	< 0.001	0.125
Helpful peers			
No suicide attempts	49.44	48.59	28.04
One or more suicide attempts	35.41	39.19	24.12
P-value	0.033	0.003	0.198

All variables are expressed as proportions (in %) with the exception of age and close friends expressed as mean and standard deviation

Table 2 adjusts for age and sex while Table 3 shows the final multivariable model. For the Cook Islands, suicide ideation, anxiety, physical bullying, and cigarette smoking were found to be statistically significantly associated with physical fighting status at p < 0.05; while at p < 0.01, only suicide ideation was found to be significant. For Curaçao, suicide ideation and suicide planning were statistically significant at p < 0.05; and the same was also found to be statistically significant at p < 0.01. For East Timor, suicide ideation, suicide planning, bullying victimization, and serious injury were statistically significant at p < 0.05; while at p < 0.01, suicide ideation, suicide planning, and serious injury were found to be significant.

**Table 2:** Outcomes of multivariable analysis of variables associated with suicide attempts adjusted for age and sex, among school-attending adolescents in Cook Islands, Curaçao, and Timor Leste, GSHS 2015

Variable	Cook Islands	Curaçao	East Timor
Age			
Adjusted OR	1.13	0.93	0.95
95% CI	0.97–1.30	0.86–1.01	0.88–1.03
P-value	0.104	0.074	0.197
Sex (male)			
Adjusted OR	0.92	0.64	1.24
95% CI	0.64–1.31	0.49–0.84	0.99–1.56
P-value	0.623	0.002	0.06
Suicide ideation			
Adjusted OR	20.88	19.25	10.51
95% CI	12.15–35.89	14.59–25.41	7.50–14.73
P-value	< 0.001	< 0.001	< 0.001
Suicide planning			
Adjusted OR	14.26	23.80	12.55
95% CI	7.62–26.70	17.44–32.49	8.70–18.12
P-value	< 0.001	< 0.001	< 0.001
Anxiety			
Adjusted OR	4.66	3.89	2.21
95% CI	2.94–7.40	2.73–5.55	1.56–3.14
P-value	< 0.001	< 0.001	< 0.001
Loneliness			
Adjusted OR	4.90	4.12	2.02
95% CI	2.47–9.72	2.98–5.70	1.34–3.05
P-value	< 0.001	< 0.001	0.002
Food deprivation			
Adjusted OR	1.51	3.77	1.68
95% CI	0.78–2.94	2.38–5.96	1.23–2.30
P-value	0.218	< 0.001	0.002
Close friends			
Adjusted OR	0.65	0.79	0.76
95% CI	0.49–0.86	0.70–0.90	0.67–0.87
P-value	0.004	< 0.001	< 0.001
Bullying victimization			
Adjusted OR	5.61	3.71	6.33
95% CI	3.54–8.91	2.78–4.96	4.27–9.39
P-value	< 0.001	< 0.001	< 0.001
Physical bullying			
Adjusted OR	4.34	3.07	4.44
95% CI	1.85–10.18	0.94–10.04	2.61–7.56
P-value	0.001	0.063	< 0.001
Parental tobacco use			
Adjusted OR	1.08	1.91	1.64
95% CI	0.73–1.60	1.48–2.46	1.24–2.17
P-value	0.701	< 0.001	0.001
People smoked in presence during the last week			
Adjusted OR	1.28	1.71	1.12
95% CI	0.82–1.99	1.29–2.26	0.88–1.42
P-value	0.269	< 0.001	0.324
Physical fight			
Adjusted OR	4.40	4.09	2.77
95% CI	2.44–7.94	2.89–5.78	1.82–4.22
P-value	< 0.001	< 0.001	< 0.001
Serious injury			
Adjusted OR	2.55	2.44	4.28
95% CI	1.59–4.09	1.78–3.34	2.88–6.36
P-value	< 0.001	< 0.001	< 0.001
Early sexual debut			
Adjusted OR	Not asked	2.49	2.47
95% CI		1.68–3.68	1.49–4.09
P-value		< 0.001	0.001
Alcohol use in the past 30 days			
Adjusted OR	1.92	2.49	4.31
95% CI	0.96–3.83	1.94–3.19	2.91–6.37
P-value	0.065	< 0.001	< 0.001
Physically attacked			
Adjusted OR	2.64	3.46	1.99
95% CI	1.65–4.23	2.38–5.05	1.50–2.66
P-value	< 0.001	< 0.001	< 0.001
Marijuana use in the past 30 days			
Adjusted OR	5.39	3.11	7.67
95% CI	3.07–9.47	2.04–4.76	5.28–11.14
P-value	< 0.001	< 0.001	< 0.001
Smoked cigarettes			
Adjusted OR	2.67	3.42	3.26
95% CI	1.24–5.71	2.38–4.91	2.32–4.58
P-value	0.013	< 0.001	< 0.001
Supportive parental figures			
Adjusted OR	0.82	0.50	1.42
95% CI	0.50–1.36	0.35–0.70	0.93–2.19
P-value	0.441	< 0.001	0.101
Helpful peers			
Adjusted OR	0.54	0.68	0.88
95% CI	0.31–0.93	0.54–0.87	0.62–1.25
P-value	0.027	0.003	0.46

All variables adjusted for age and sex, while age and sex were adjusted for each other. OR is odds ratio and 95% CI is 95% confidence intervals

**Table 3:** Outcomes of multivariable analysis of variables associated with suicide attempts among school-attending adolescents in Cook Islands, Curaçao, and Timor Leste, GSHS 2015

Variable	Cook Islands	Curaçao	East Timor
Age			
Adjusted OR	NA	NA	NA
95% CI			
P-value			
Sex			
Adjusted OR	NA	0.83	NA
95% CI		0.53–1.31	
P-value		0.431	
Suicide ideation			
Adjusted OR	19.42	7.88	4.59
95% CI	9.11–41.41	5.20–11.95	2.14–9.88
P-value	< 0.001	< 0.001	0.001
Suicide planning			
Adjusted OR	2.51	7.01	3.36
95% CI	0.97–6.52	4.24–11.60	1.79–6.29
P-value	0.057	< 0.001	0.001
Anxiety			
Adjusted OR	2.51	1.12	2.11
95% CI	1.08–5.82	0.58–2.19	0.95–4.69
P-value	0.033	0.726	0.066
Loneliness			
Adjusted OR	0.43	1.55	0.98
95% CI	0.09–1.98	0.96–2.51	0.45–2.16
P-value	0.272	0.071	0.963
Food deprivation			
Adjusted OR	NA	2.14	1.31
95% CI		0.95–4.81	0.58–2.97
P-value		0.065	0.497
Close friends			
Adjusted OR	0.93	0.997	0.78
95% CI	0.62–1.40	0.82–1.21	0.59–1.03
P-value	0.735	0.979	0.073
Bullying victimization			
Adjusted OR	1.81	1.57	2.69
95% CI	0.51–6.35	0.92–2.66	1.02–7.12
P-value	0.347	0.094	0.047
Physical bullying			
Adjusted OR	3.3	NA	0.64
95% CI	1.10–9.91		0.12–3.31
P-value	0.034		0.573
Parental tobacco use			
Adjusted OR	NA	1.31	0.84
95% CI		0.84–2.06	0.45–1.57
P-value		0.235	0.562
People smoked in presence during the last week			
Adjusted OR	NA	1.05	NA
95% CI		0.67–1.66	
P-value		0.827	
Physical fight			
Adjusted OR	2.86	1.92	1.31
95% CI	0.93–8.81	0.97–3.82	0.61–2.82
P-value	0.067	0.062	0.473
Serious injury			
Adjusted OR	1.15	1.24	2.22
95% CI	0.58–2.30	0.74–2.08	1.31–3.74
P-value	0.684	0.413	0.005
Early sexual debut	Not asked		
Adjusted OR		1.01	0.99
95% CI		0.60–1.72	0.33–2.95
P-value		0.969	0.986
Alcohol use in the past 30 days			
Adjusted OR	0.60	1.28	1.19
95% CI	0.23–1.55	0.88–1.87	0.61–2.33
P-value	0.287	0.193	0.597
Physically attacked			
Adjusted OR	0.79	1.39	1.19
95% CI	0.24–2.58	0.86–2.25	0.65–2.20
P-value	0.689	0.172	0.550
Marijuana use in the past 30 days			
Adjusted OR	1.62	1.12	1.79
95% CI	0.50–5.19	0.42–2.95	0.68–4.69
P-value	0.411	0.823	0.224
Smoked cigarettes			
Adjusted OR	3.82	1.19	1.33
95% CI	1.38–10.54	0.55–2.56	0.78–2.27
P-value	0.011	0.659	0.272
Supportive parental figures			
Adjusted OR	NA	0.95	NA
95% CI		0.58–1.56	
P-value		0.847	
Helpful peers			
Adjusted OR	0.39	0.84	NA
95% CI	0.15–1.00	0.57–1.24	
P-value	0.05	0.387	

Only those factors found statistically significant in bivariate analysis were used in this model. OR is odds ratios, 95% CI is 95% confidence intervals

## Discussion

An inquiry into the mental health and health behaviors in nations with young populations, as in the context of the current research, has implications for economic and social advancement. The rates of SA among in-school adolescents in the Cook Islands (13.6%), Curaçao (10.8%), and East Timor (9.8%) were similar to the reports from many high-income countries [16] but higher than those from the USA and Canada [17]. The rates were, however, lower than those of sub-Saharan Africa [17]. Differences in income levels and standards of living might explain this variation [18]. Stable economic and social settings may mitigate the expression of unhealthy behavior among young people. The attributes of in-school adolescents in the three island nations are similar despite their diverse history, culture, economic, and social contexts. The islands have a similar age and sex distribution, and marijuana use and physical bullying were low. However, the individual nations had some distinctive characteristics. Tobacco use, physical attack, and food deprivation were more prevalent in East Timor and the Cook Islands than in Curaçao. Also, early sexual debut, alcohol use, and supportive parental figure were more common in Curaçao relative to the other two Islands. Curaçao is a high-income economy with a higher standard of living. It is ranked 27th in the world in terms of the Gross Nominal Domestic Product per capita (nGDP per capita). Curaçao’s nGDP per capita of United States Dollars (USD) 47,020 far exceeds those of the Cook Islands (USD 17,797), and East Timor (USD 2422) [19–22]. Previous studies have suggested negative and positive adolescent attributes are disproportionately distributed in high and low- and middle-income countries (LMICs) [23–27].

The association of adolescent suicidal ideation and planning with suicidal attempt corroborates findings from diverse other countries and contexts [16, 28–31]. Nevertheless, suicidal ideation does not always result in suicide attempts [32, 33]. With prevention in mind it is crucial to investigate mechanisms facilitating the progression from ideation to attempt. Many hypotheses on this topic exist. The three-step theory (3ST) represents contemporary understanding within the ideation-to-attempt framework, pointing out that the capacity for SA, consisting of dispositional, acquired, and practical variables enabling the SA capability, is the main factor for transitioning from suicidal ideation to attempt. This is consistent with the interpersonal theory of suicide (IPTS). The IPTS additionally indicates the contribution of the exposure to painful and provocative events (PPEs), leading to habituation to pain and fear, to SA capacity. PPEs may also explain the significant association between SA and physical bullying and serious injury shown in our results. The lack of association between suicide planning and SA in the Cook Islands is surprising and would benefit from further exploration to determine the responsible contextual factors. Also, in the Cook Islands, anxiety, physical bullying, and smoking are associated with SA, while helpful peers are protective. These findings align with reports of previous studies [16, 18, 31, 34–36].

Although there was no significant association between SA and involvement in physical fights or being physically attacked, bullying was associated in Cook Islands. In East Timor, we found that bullying victimization and serious injuries had a statistically significant association with SA in keeping with previous findings [31, 36]. Previous studies have researched different forms of bullying, including verbal harassment, physical aggression, and cyberbullying, and which were all suggested to be associated with suicidal behavior [37–41]. However, Kodish et al. pointed out that when only SA was considered, only verbal bullying showed association [37]. It is possible that the emotional distress caused by power imbalance and being isolated among peers, not necessarily physical injury, is linked with SA as far as bullying is concerned. Adolescents’ lack of experience in coping with interpersonal conflicts and emotional fluctuations can contribute to impulsive and self-destructive behaviors as well. Furthermore, other researches indicate that mediators like depression, anxiety, low self-esteem, loneliness, and hopelessness, affect the association between bullying and suicidal behaviors during adolescence. These mediators impact directly on psychological health, but may also result from mental ill-health [34]. The victims of bullying may perceive a lack of safety and belonging with reduced social support in the school environment, potentially heightening anxiety and loneliness. In a study of young adults aged 14 to 24 years, anxiety disorder was identified as the prime risk factor for SA among various other illnesses [42, 43]. Loneliness is associated with an increased risk of substance abuse [44].

In the Cook Islands, cigarette smoking had a significant association with SA. In a 2016 meta-analysis, a significant association between current smoking and suicidal behaviors was found [45]. The cumulative results indicated parental tobacco use and cigarette smoking have a low prevalence in Curaçao, while both factors showed high prevalence in the Cook Islands and Timor Leste, where almost half of the respondents have experienced people smoking in their presence in the preceding 7 days. In a way, the smoking behaviors of the youths might have been influenced by that of family members and significant others. Measures are required to strengthen awareness among the parents on how their behaviors influence young people.

Gender had no statistically significant relationship with SA. This finding contradicts existing literature demonstrating that SA and gender are associated, with most studies suggesting the males are more likely to attempt suicide and self-harm [18, 46]. However, a few authors have reported the opposite [16, 47, 48]. Early sexual debut did not play a role in SA in the three Islands under consideration, although it showed a high correlation in Brunei and Malawi [16, 31]. These differences highlight the probable effect of context in moderating the factors that underlie SA. An in-depth understanding of adolescents’ characteristics and socioeconomic circumstances should precede policy-making, strategy formulation, and program implementation concerning SA.

## Conclusion

Despite population and socioeconomic differences between the studied countries, SA is common with similar rates in each country. Adolescents in the three Islands had a disproportionate distribution negative attributes potentially influenced by differences in living standards in each country. These differences highlight the need to investigate the effect of wider social, environmental and economic contexts outside of school environments.

## Limitations

The cross-sectional nature of these data are not amenable to causal interpretations. Secondly, as these are self-reported responses, they are subject to social desirability bias. Lastly, the lack of responses from adolescents who were absent from school on the day of the survey.

## Supplementary information

**Additional file 1:** Table S1: Independent variable derivation from GSHS survey data (Cook Islands, Curacao, East Timor) 2015.

**Additional file 2:** Table S2: Cumulative proportion of factors in school-attending adolescents in Cook Islands, Curacao, and East Timor, GSHS 2015.

## Data Availability

The datasets supporting this analysis are publicly available at http://www.cdc.gov/gshs/.

## Abbreviations

LMIC: Low- and middle-income countries
SA: Suicide attempts
CI: Confidence intervals
nGDP: Gross Nominal Domestic Product per capita
USD: United States Dollars
3ST: Three-step theory
IPTS: Interpersonal theory of suicide
PPE: Painful and provocative events
